# Adverse drug reactions to the three doses of the severe acute respiratory syndrome coronavirus 2 (SARS-COV-2) mRNA-1273 vaccine in a cohort of cancer patients under active treatment of a tertiary hospital in Madrid, Spain

**DOI:** 10.12688/f1000research.110268.1

**Published:** 2022-04-19

**Authors:** Javier David Benitez Fuentes, Alicia de Luna Aguilar, Alejandro Francisco Jimenez Ortega, Paloma Flores Navarro, Jorge Bartolomé Arcilla, Elvira Baos Muñoz, Alberto Delgado-Iribarren García-Campero, Sara Gil Useros, Ignacio Martinez Capella, Laura Llorente Sanz, Macarena Torrego Ellacuría, Pedro Pérez Segura

**Affiliations:** 1 Medical Oncology Department, IdISSC, Hospital Clinico San Carlos, Madrid, Madrid, 28040, Spain; 2 Bloomberg School of Public Health MD, 21205, USA, Johns Hopkins University, Baltimore, Maryland, 21205, USA; 3 Clinical Pharmacology Department, IdISSC, Hospital Clinico San Carlos, Madrid, Madrid, 28040, Spain; 4 Clinical Microbiology Department, IdISSC, Hospital Clínico San Carlos, Madrid, Madrid, 28040, Spain; 5 Innovation Unit, IdISSC, Hospital Clínico San Carlos, Madrid, Madrid, 28040, Spain

**Keywords:** COVID-19, mRNA-1273 Vaccine, SARS-CoV-2, Safety, Cancer, Oncology

## Abstract

**Background: **Severe acute respiratory syndrome coronavirus 2 (SARS-CoV-2) vaccines efficacy and safety have been tested in phase 3 studies in which cancer patients were not included or were underrepresented.

**Methods:** The objective of this study is to evaluate the safety profile of the mRNA-1273 vaccine across cancer patients and its relationship to patients’ demographics. This retrospective cohort study included patients 18-years or older with solid malignancies receiving active treatment in our hospital who had received the three-dose schedule of the mRNA9 1273 vaccine and whose side effects after each dose were recorded. Patient electronic medical records were reviewed retrospectively to collect data between April 19, 2021, and December 31, 2021. Patients with documented previous infection by SARS-Cov-2 were excluded from the study.

**Results:** A total of 93 patients met the inclusion criteria. Local adverse drug reactions (ADRs) were reported more frequently after the first and second dose than after the third (41.9%, 43% and 31.1% of the patients respectively), while systemic ADRs followed the opposite pattern (16.1%, 34.4% and 52.6% of the patients respectively). We found a statistically significant association between sex and systemic ADRs after the third dose. Cochran-Armitage test showed a statistically significant linear trend, *p* = 0.012, with a higher Eastern Cooperative Oncology Group (ECOG) score associated with a lower proportion of patients suffering from systemic side effects. A logistic regression showed that women had 5.79 times higher odds to exhibit systemic ADRs after the third dose (p=0.01) compared to males. Increasing age was associated with a decreased likelihood of exhibiting ADRs (p=0.016).

**Conclusion:** The mRNA-1273 vaccine shows a tolerable safety profile. The likelihood of ADRs appears to be associated with gender and age. Its association with ECOG scores is less evident. Further studies are needed to elucidate this data in cancer patients.

## Introduction

In December 2019, cases of pneumonia of unknown aetiology related to a seafood market were found in Wuhan, China. A previously unknown betacoronavirus was isolated from human epithelial cells, it was named severe acute respiratory syndrome coronavirus 2 (SARS-CoV-2) and the disease it causes coronavirus disease 2019 (COVID-19). The virus spread exponentially through Hubei province in China, affecting the whole country afterwards and then the rest of the world. ^1^ In January 2020 the World Health Organization (WHO) declared an international emergency after the expansion of the virus to other countries, infecting 10,000 people and provoking 200 deaths. ^2^ On 11th March, following a further increase in the number of cases outside China and the number of countries affected, the WHO declared COVID-19 a pandemic. ^3^ COVID-19 has shown a wide variety of symptoms and a broad spectrum of severity. ^4^ SARS-CoV-2 infection has been associated with worse clinical outcomes in patients with cancer, with an estimated mortality of 30% in hospitalized cancer patients and 60% in cancer patients admitted to the Intensive Care Unit. ^5^^–^^7^ Patient care in this population was disrupted during the pandemic due to the emergency situation. In many cases surgeries and cancer medical treatments were delayed to prevent cancer patients from getting the infection. ^8^ Due to the emergency generated by the pandemic several research projects involving vaccines against SARS-CoV-2 were started. In December 2020 the Food and Drug Administration (FDA) issued the first Emergency Use Authorization for the BNT162b2 vaccine after it was found to be safe and efficient in preventing COVID-19 in the general population, followed shortly by the mRNA-1273 vaccine. ^9^ Patients receiving systemic immunosuppressants or immune modifying drugs within six months of screening were excluded from the clinical trials, ^10^^,^^11^ thus leaving cancer patients behind in the research for a vaccine against SARS-CoV-2. However, despite the lack of evidence in this population, cancer patients were prioritized for the administration of the SARS-CoV-2 vaccine. ^12^ In Spain cancer patients started vaccination in April 2021.

There are few studies showing the safety of mRNA-1273 vaccine in solid cancer patients.

In this study we describe and analyse the safety profile of the mRNA-1273 vaccine in a cohort of 93 cancer patients in cancer treatment in a tertiary hospital in Madrid, Spain.

## Methods

### Ethical considerations

The Comité de Ética del Medicamento e Investigación Clínica (Ethics Committee) of Hospital Clínico San Carlos approved the project with the code: 22/033-E. The Comité de Ética del Medicamento e Investigación Clínica (Ethics Committee) of Hospital Clínico San Carlos deemed the necessary requirements for appropriateness of the protocol in relation to the objectives of the study were met, the informed consent waiver was considered adequate, the procedure foreseen for the handling of personal data was adequate. The ethical precepts formulated in the Declaration of Helsinki of the World Medical Association for medical research on human beings and its subsequent revisions are complied with, as well as those required by the applicable legal regulations according to the characteristics of the study.

### Study design, eligibility and study procedures

This observational retrospective study included patients with solid tumours receiving anticancer treatment at the outpatient facility of the Hospital Clínico San Carlos Medical Oncology service in Madrid, Spain. We included all the patients vaccinated with the complete three-dose schedule mRNA-1273 vaccine that were on active anticancer therapy and had complete available information about the date of each vaccination dose and side effects for each of the three doses in electronic medical records.

We selected the patients vaccinated with the complete three-dose schedule mRNA-1273 vaccine from the Preventive Medicine and Public Health Department database. This database was linked with the Medical Oncology Department patient database selecting all oncology patients under active treatment who were in the previous database. From these subjects we selected the ones that had information about the appearance or absence of adverse drug reactions (ADRs) after each dose of the vaccine available in electronic medical records. Patients who had a documented SARS-CoV-2 infection or a positive SARS-CoV-2 serology test collected during routine clinical practice in the 7 days prior to the first mRNA-1273 vaccine dose were excluded.

Additional clinical information was abstracted from the electronic medical records, including age, sex, performance status (using Eastern Cooperative Oncology Group (ECOG) performance status score), ^13^ cancer type, cancer stage and cancer therapy. Patient electronic medical records were reviewed retrospectively to collect the information regarding the previous parameters between the dates April 19, 2021, and December 31, 2021. Authors belonging to Medical Oncology Department had complete access to all electronic medical records available from the patients.

ECOG performance status score describes the level of functioning in terms of the ability to selfcare, daily activity, and physical ability of the patients. ECOG 0 patients are fully active. ECOG 1 patients are not able to perform physically demanding activity but are ambulant and able to perform occupations of light nature. ECOG 2 patients are ambulant, up for more than 50% of waking hours, and capable of all personal care, but are unable to perform any work activities. ECOG 3 patients can perform only limited self-care and are bedridden or confined to a chair for more than half of waking hours. ECOG 4 patients are completely incapacitated, unable to perform any self-care and totally confined to a bed or chair. ECOG 5 patients are dead. ^13^

Cancer type was divided in seven groups: thoracic malignancies, breast cancer, head and neck cancer, gastrointestinal malignancies, gynaecological malignancies, and others (malignancies that did not belong to any of the previous groups).

Cancer stage was divided in metastatic, patients with malignant lesions in locations organs distant from where the primary tumour is, and the rest, defined as localized.

Cancer therapy was divided in chemotherapy, targeted therapy, immunotherapy and combined therapy (any combination of the previous treatments).

### Data analysis

The primary end point of this study was ADRs after each dose of mRNA-1273 vaccine. ADRs were categorized as local adverse reactions which included pain, swelling, rash and itchiness at the site of infection and systemic adverse reactions. Systemic adverse reactions reported by patients were fever (defined as body temperature equal or above 38°C), headache, myalgia, malaise, nausea, arthralgia, chills, adenopathies, urticaria, asthenia and cough.

Data was analysed with IBM SPSS v.25. For descriptive purposes, categorical variables were represented by absolute and relative frequencies and quantitative variables were represented by central and dispersion measures. In order to ascertain the relationship between nominal independent variables (sex, heavily treated status and past story of systemic adverse drug reactions) we performed a chi-squared test (all expected cell frequencies were greater than five) followed by a Cramér’s V test. For ordinal variables (ECOG performance status score) ^14^ we performed a Cochran-Armitage test of trend. Significance was tested with an alpha value of 0,05. No multiplicity correction was applied. To test the value as predictors of true baseline variables (age, sex and ECOG performance status) we fitted a binomial logistic regression to ascertain the effects of age, sex and ECOG score on the likelihood that participants have systemic adverse events after the third dose (hereinabove described). We used a Box-Tidwell procedure to evaluate the linear relationship between the logit of the outcome and continuous variables. Following a Bonferroni correction, statistical significance was accepted hen p < 0.0071. Age was included as a continuous variable, sex as a dichotomous variable, being male considered as reference, and ECOG scores were included as categorical variables, with a score of 0 considered the reference.

## Results

Data was retrieved from electronic medical records on the 31 ^st^ of December. In total 93 patients were eligible for the current analysis. ^18^ Patient demographic, cancer, and therapy characteristics included are summarized in Table 1.

**Table 1.** Demographic, cancer, and therapy characteristics of the patients.

Characteristic	N = 93
Age, years (SD)	61 [SD 8]
Sex, No. (%)	
Female	62 (66.6)
Male	31 (33.3)
Cancer type, No. (%)	
Thoracic	11 (11.8)
Breast	32 (34.4)
Head and Neck	9 (9.7)
Gastrointestinal	16 (17.2)
Genitourinary	8 (8.6)
Gynaecological	10 (10.8)
Other	7 (7.5)
Stage, No. (%)	
Localised	31 (33.3)
Metastatic	62 (66.6)
Treatment modality, No. (%)	
Chemotherapy	34 (36.6)
Targeted therapy	21 (22.6)
Immunotherapy	11 (11.8)
Combined therapy (any combination of the previous treatments)	27 (29)
Heavily treated (3 or more lines of previous treatment), No. (%)	
Yes	29 (31.2)
No	64 (68.8)
ECOG, No. (%)	
0	55 (59.1)
1	30 (32.3)
2	8 (8.6)

SD: Standard Deviation; ECOG: East Cooperative Oncology Group Performance Status Scale. ECOG 0: fully active. ECOG 1: not able to perform physically demanding efforts but are ambulant and capable of performing tasks of sedentary nature. ECOG 2: outpatient, fully capable of all personal care but unfit for any working activity. Up for more than half of waking time.

**Figure 1.**
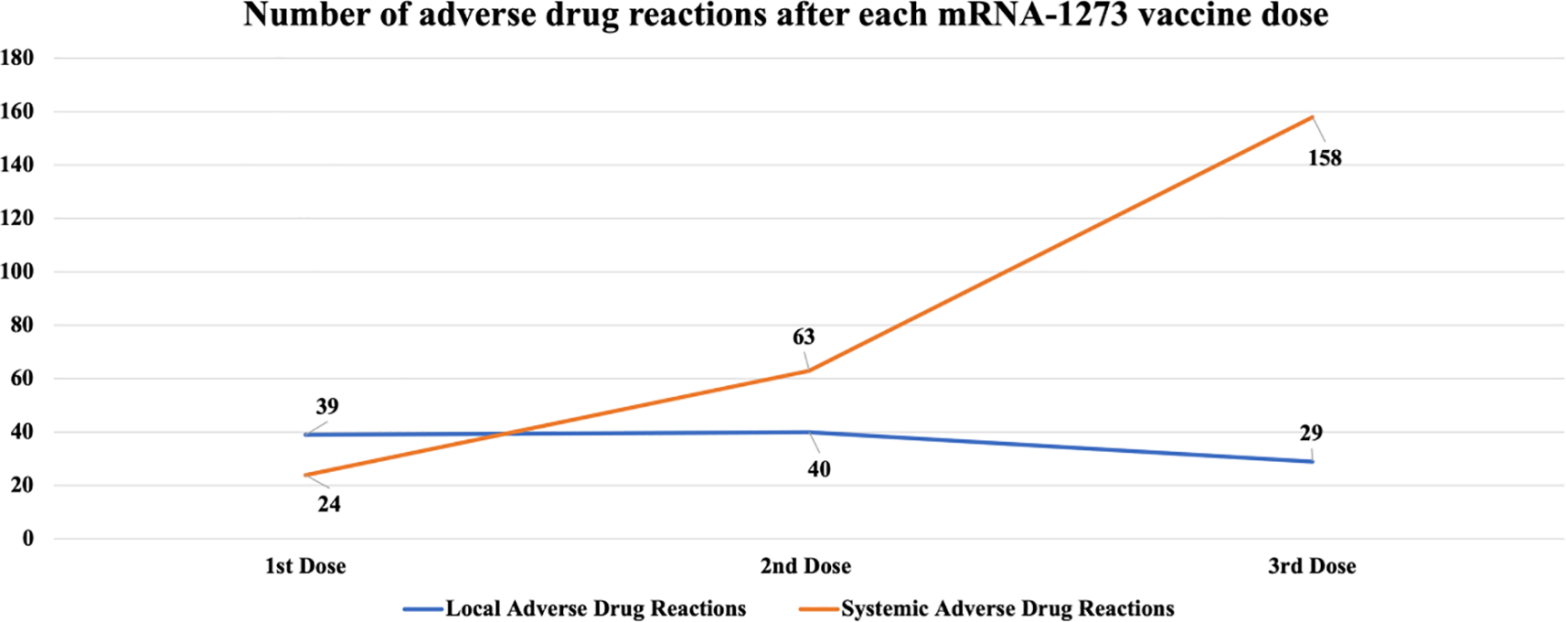
Number of ADRs after each mRNA-1273 vaccine dose.

The number of ADRs categorized as local and systemic after the first, the second and the third vaccine dose are shown in Figure 1. Local ADRs included pain, swelling, rash and itchiness at the site of infection. We can observe that systemic ADRs have a clearly increasing trend while local ADRs have a discrete downward trend.

After the first, second and third dose 41.9%, 43% and 31.1% of the patients respectively reported a local ADR, while systemic ADRs were increasingly reported after each dose (16.1%, 34.4% and 52.6% of the patients respectively).

**Figure 2.**
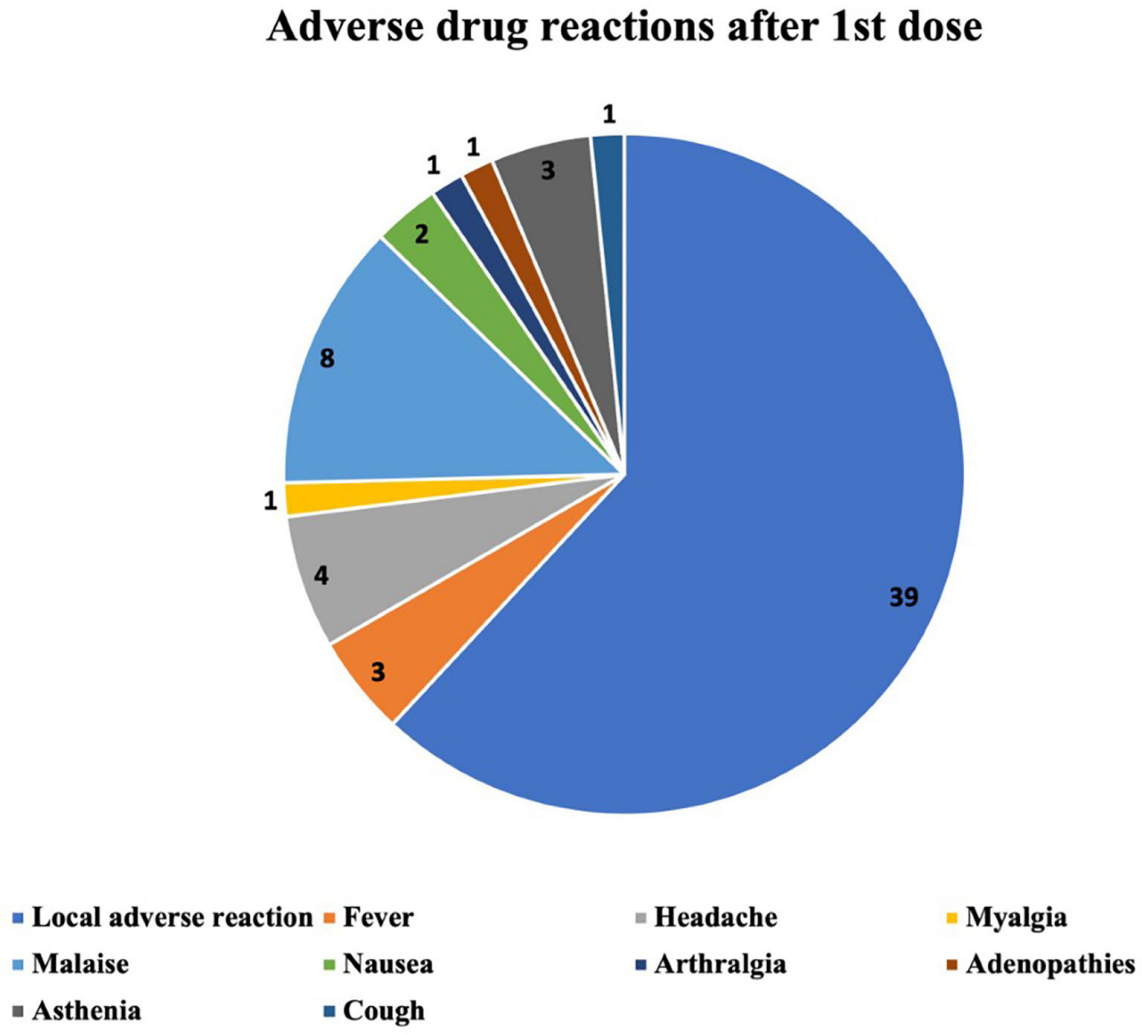
Number and type of adverse drug reactions after 1st dose.

**Figure 3.**
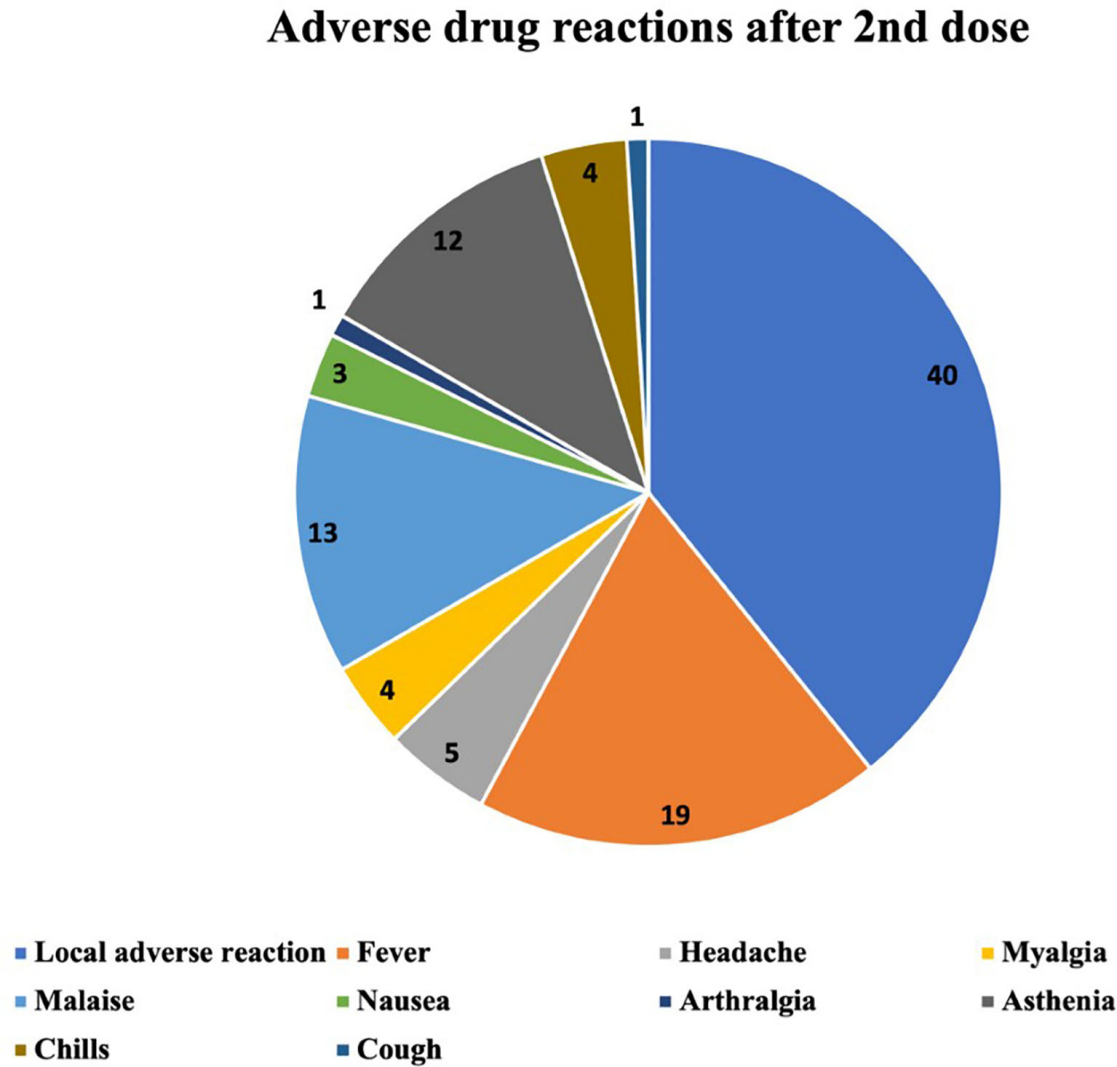
Number and type of adverse drug reactions after 2nd dose.

**Figure 4.**
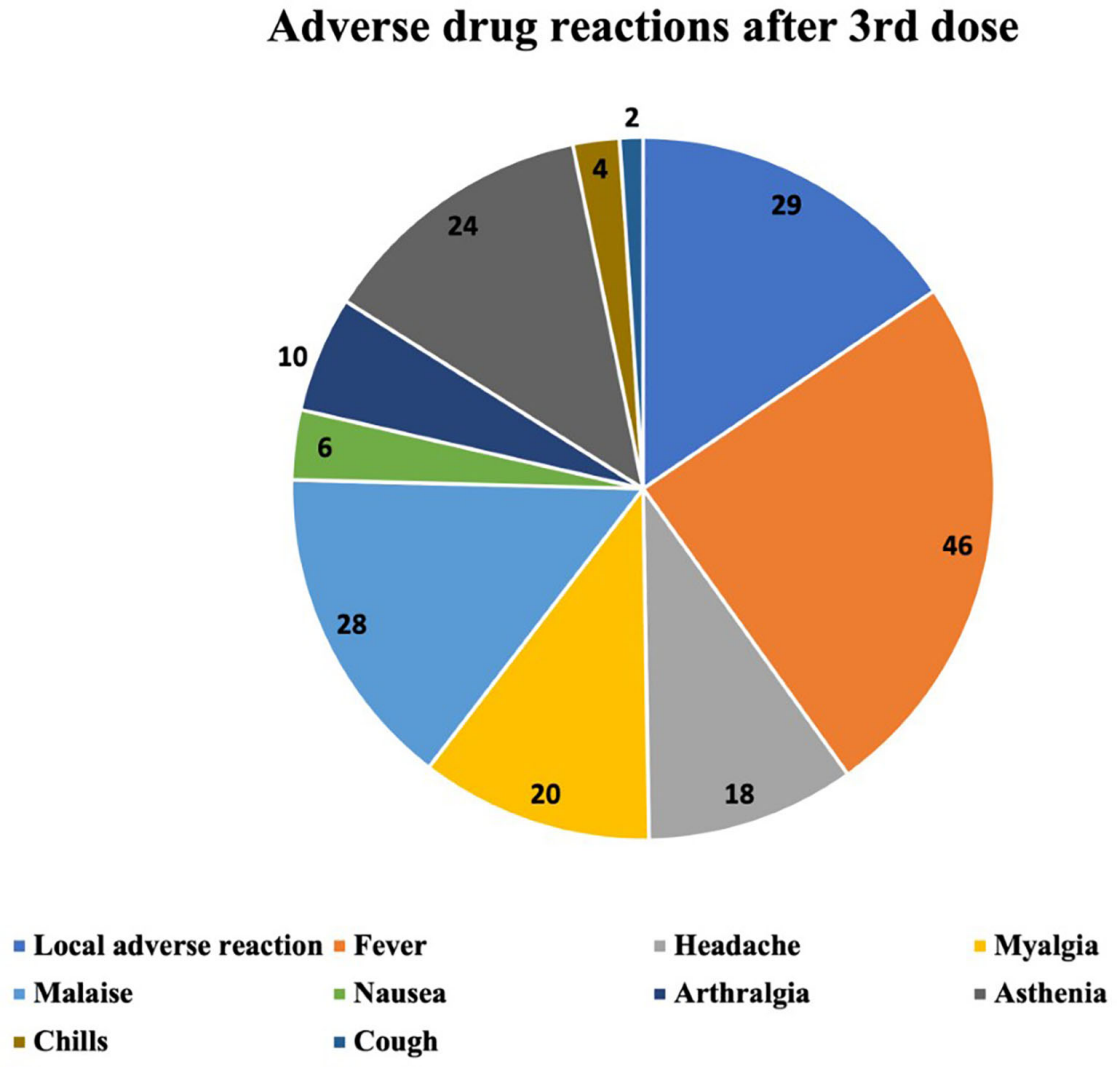
Number and type of adverse drug reactions after 3rd dose.

The ADRs occurring after the first, the second and the third vaccine dose are described in Figures 2– 4 respectively. The most common ADR after the first dose was local reaction with a marked difference compared with systemic ADRs. After the second dose the most frequent ADR was still local reaction, but systemic were becoming increasingly important. After the third dose we can observe that local ADR is less common and takes second place after fever and that the rest of systemic effects are increasingly frequent.

No severe ADRs requiring hospitalization occurred in this population. No new SARS-CoV-2 infections occurred in this population during the period studied.

There was a statistically significant association between sex and systemic adverse reactions after the third dose, χ ^2^(1) = 14.620, p ≤ 0.01. The association was moderate (Cramer's V = 0.390). We didn’t find a statistically significant association between heavily treated status and systemic adverse reactions after the third dose, χ ^2^(1) = 0.063, p = 0.802. The association was small (Cramér's V = 0.05).

Regarding adverse reactions after previous vaccine doses, there was not a statistically significant association between systemic adverse reactions after the first dose and after the third one, χ ^2^(1) = 1.897, p = 0.168, and the association between the variables small, Cramér's V = 0.172. On the other hand, we found a statistically significant association between systemic adverse reactions after the second dose and systemic adverse reactions after the third dose, χ ^2^(1) = 12.886, p < 0.001. The association was moderate, Cramér's V = 0.372.

The Cochran-Armitage test of trend showed a statistically significant linear trend, p = 0.012, with a higher ECOG score associated with a lower proportion of patients suffering from systemic side effects. The scores tested were ECOG 0 (n = 56), ECOG 1 (n = 30), ECOG 2 (n = 7), and the proportion of patients suffering a side effect was 0.643, 0.4 and 0.286, respectively.

**Figure 5.**
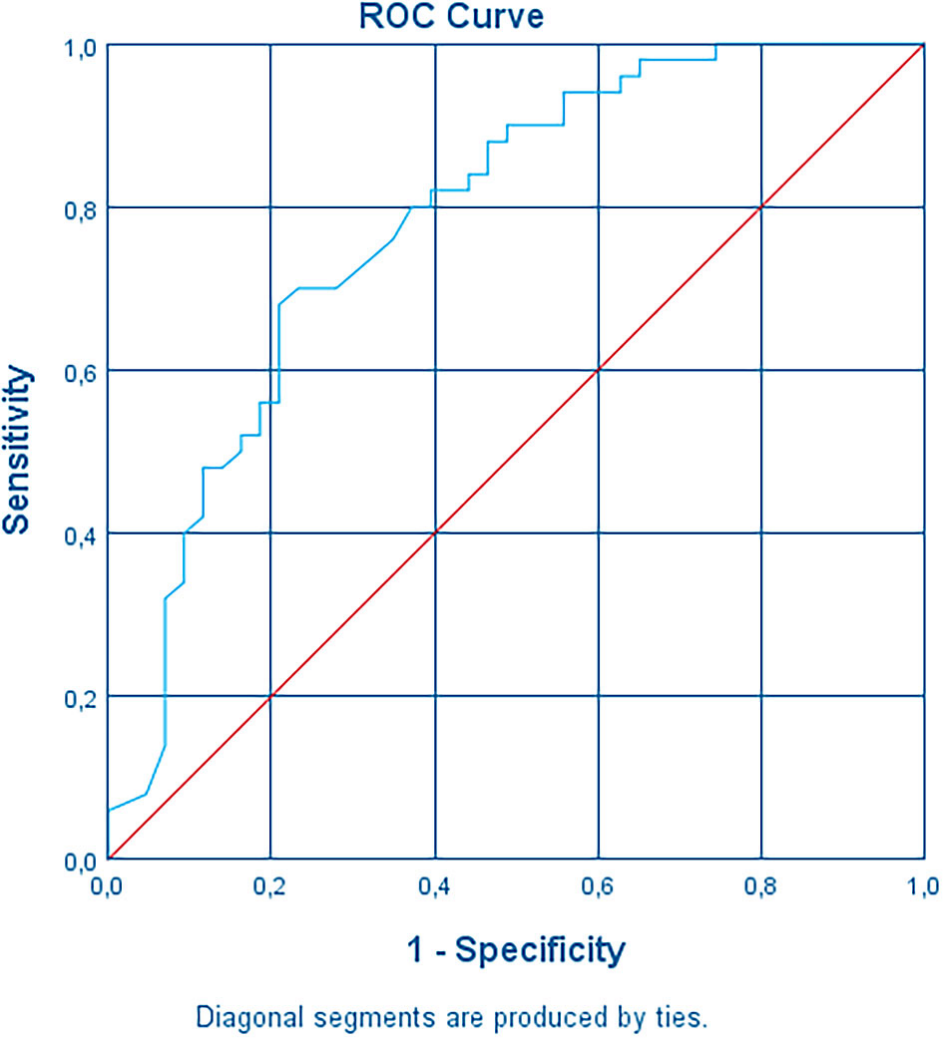
Receiving Operating Characteristic curve (ROC curve).

Age was found to be linearly related to the logit of the dependent variable. The area under the ROC curve was 0.783 (95% CI, 0.688 to 0.878) ( Figure 5), which is at the upper level of an acceptable level of discrimination according to. ^16^ The logistic regression model was statistically significant, χ ^2^(4) = 25.641, p < .0005. The model explained 32.2% (Nagelkerke R2) of the variance in systemic adverse events after the third dose and correctly classified 72.0% of cases. Sensitivity was 82%, specificity was 60.5%, positive predictive value was 70.6% and negative predictive value was 74.2%.

Only two variables were statistically significant: age and sex (as shown in Table 2). Women had 5.79 times higher odds to exhibit systemic adverse events after the third dose compared to males. Increasing age was associated with a decreased likelihood of exhibiting adverse events. ECOG score was not statistically significant but a trend towards a diminished likelihood of systemic adverse reactions with higher ECOG score compared to ECOG 0.

**Table 2.** Logistic regression predicting Likelihood of Systemic Adverse Reactions based on Age, Sex and ECOG score.

	B	SE	Wald	df	*p*	OR	95% CI for OR	
							Lower	Upper
**ECOG 1**	-,434	,538	,652	1	,420	,648	,226	1,858
**ECOG 2**	-1,669	,967	2,979	1	,084	,188	,028	1,254
**Female Sex**	1,756	,533	10,839	1	,001	5,789	2,035	16,468
**Age (years)**	-,059	,024	5,804	1	,016	,943	,899	,989
**Constant**	2,846	1,524	3,485	1	,062	17,215		

Abbreviations: B logarithmic regression slope, SE standard error, degrees of freedom, OR odds ratio, CI confidence interval. Regarding the variable Sex, male was considered the reference. Regarding the variable ECOG, ECOG 0 was considered the reference.

## Discussion

Among completed and ongoing trials of SARS-CoV-2 vaccines there is scarce information regarding safety and efficacy of these vaccines in solid cancer patients. Patients receiving systemic immunosuppressants or immune modifying drugs within six months of screening were excluded from the major vaccine trials. ^9^^,^^11^

In the phase 3 clinical trial of the mRNA-1273 SARS-CoV-2 vaccine, the mRNA-1273 group showed 84.2% of local ADRs after the first dose and 88.6% after the second dose. Systemic ADRs in the mRNA-1273 group occurred in 54.9% of patients after the first dose and 79.4% of patients after the second. Fever, headache, and myalgia were the most common systemic ADRs. ^11^ In our population we observed both less local and systemic ADRs than in the healthy population from this trial.

Only a few studies report on the safety profile of the COVID-19 vaccines in cancer patients, fewer report data specifically about the mRNA-1273 vaccine safety profile in this population, and none of them about the third dose.

In a study from the Massachusetts General Hospital Cancer Center, in patients vaccinated with mRNA-1273, BNT162b2 and Ad26.COV2.S, the frequency of local or systemic ADRs was highest in the mRNA-1273 recipients; 233 of 288 (81%) had an ADR. Systemic ADRs were more common after the second dose of the vaccine. The most common systemic ADR was fatigue. ^15^

In another study from the Netherlands four cohorts were studied, one made of individuals without cancer (cohort A), cancer patients treated with immunotherapy (cohort B), cancer patients treated with any type or combination of cytotoxic chemotherapy (cohort C) and cancer patients treated with chemoimmunotherapy (cohort D). No major adverse events of grade 3 or worse happened in any participant in cohort A. In the rest of the cohorts, serious adverse events occurred in 2% of 137 patients in cohort B, in 2% of 244 patients in cohort C, and in 1% of 163 patients in cohort D. Only four events (two of fever and one each of diarrhoea and febrile neutropenia) were potentially related to vaccination. Systemic ADRs were more common after the second dose with myalgia, headache, chills, and fatigue being the most common. No vaccine related deaths were reported. ^16^

In another study from Italy in patients with solid malignancies undergoing radiotherapy treatment, 5% of patients after the first dose and 26% after the second dose reported grade 2 ADRs. After the first dose 0% reported grade 4 ADRs and only 4% reported them after the second dose. In this study we can also observe the increasing trend of systemic ADRs with the second dose. ^17^

In all these studies we observe an increasing trend of systemic ADRs. Our study is, to our knowledge, the only one evaluating the safety profile of each of the three doses of mRNA-1273 vaccine in cancer patients. Our study confirms the increasing frequency of systemic ADRs after each dose. In a scenario where the start of the administration of the fourth dose in cancer patients is near, we expect that this trend will be maintained in the fourth dose. We have also observed an association of ADRs with gender and age, which could help us predict subgroups at greater risk of ADRs in the event of more doses of the vaccine.

This study has some important limitations. (1) The retrospective nature of the study makes it more prone to error, making measurements less precise. (2) The small population in this study and its characteristics do not represent the general cancer patient population and makes it difficult to generalize the results, thus external validity can be compromised. (3) The absence of a healthy control group makes it difficult to reach conclusions.

## Conclusion

Although based on a small number of patients and limited by the observational nature of the study, the mRNA-1273 vaccine shows a tolerable safety profile in this cohort of cancer patients similar to the non-oncologic population. No severe ADRs requiring hospitalization occurred in this population. The likelihood of ADRs appears to be associated with gender and age. The likelihood of systemic ADRs after the third dose appears to be associated with systemic ADRs after the second dose. There appears to be a trend towards more systemic and less local ADRs with every consecutive dose of the vaccine. Its association with ECOG performance score is less evident. To date this is the first study evaluating the safety profile of the three doses of mRNA-1273 vaccine.

## Data availability

### Underlying data

Dryad: Adverse drug reactions to the three doses of the SARS-COV-2 mRNA-1273 vaccine in a cohort of cancer patients of a tertiary hospital. https://doi.org/10.5061/dryad.cnp5hqc6d. ^18^

This project contains the following underlying data:

• ADRs_mRNA-1273_database_excel.xlsx (database with all the individual anonymized data regarding the parameters analysed in this article)
• README.docx (document with the explanation of the data used in the database)t

Data are available under the terms of the Creative Commons Zero “No rights reserved” data waiver (CC0 1.0 Public domain dedication).
