# Supplementary material for: Adverse drug reactions to the three doses of the severe acute respiratory syndrome coronavirus 2 (SARS-COV-2) mRNA-1273 vaccine in a cohort of cancer patients under active treatment of a tertiary hospital in Madrid, Spain
Source: F1000Res. 2022 Jul 26;11:434. Originally published 2022 Apr 19. [Version 2] doi: 10.12688/f1000research.110268.2 (PMC9816496; doi:10.12688/f1000research.110268.2)
Supplement: Peer review history

# Peer review: Reviewer response for version 2

**DOI:** 10.5256/f1000research.136429.r157681

**Published:** 2023-01-05

**Authors:** Durga Neupane

**Affiliations:** 1 Department of Surgery, B.P. Koirala Institute of Health Sciences, Dharan, Nepal

First of all, thank you for giving me an opportunity to review this article. The authors have done a good study about the safety and efficacy of COVID vaccine targeted on solid cancer patients. The former reviewer's comments have been addressed properly. I recommend that the article is copy edited. Otherwise, it has added significantly to existing literature. I recommend that it is indexed without any revision.

 Thank you.

Is the work clearly and accurately presented and does it cite the current literature?

Yes

If applicable, is the statistical analysis and its interpretation appropriate?

Yes

Are all the source data underlying the results available to ensure full reproducibility?

Yes

Is the study design appropriate and is the work technically sound?

Yes

Are the conclusions drawn adequately supported by the results?

Yes

Are sufficient details of methods and analysis provided to allow replication by others?

Yes

Reviewer Expertise:

Neurosurgery; Oncology; Public Health; Neuroscience

I confirm that I have read this submission and believe that I have an appropriate level of expertise to confirm that it is of an acceptable scientific standard.

# Peer review: Reviewer response for version 2

**DOI:** 10.5256/f1000research.136429.r145418

**Published:** 2022-08-30

**Authors:** Manushak Avagyan

**Affiliations:** 1 International Agency for Research on Cancer, Lyon, France; 2 Hematology Center after Prof. Yeolyan, Yerevan, Armenia

Thank you for improving the manuscript further.

 There are no additional remarks.

Is the work clearly and accurately presented and does it cite the current literature?

Partly

If applicable, is the statistical analysis and its interpretation appropriate?

I cannot comment. A qualified statistician is required.

Are all the source data underlying the results available to ensure full reproducibility?

Yes

Is the study design appropriate and is the work technically sound?

Yes

Are the conclusions drawn adequately supported by the results?

Yes

Are sufficient details of methods and analysis provided to allow replication by others?

Yes

Reviewer Expertise:

Childhood cancer, cancer epidemiology, childhood cancer registration, cancer registries.

I confirm that I have read this submission and believe that I have an appropriate level of expertise to confirm that it is of an acceptable scientific standard.

# Peer review: Reviewer response for version 1

**DOI:** 10.5256/f1000research.121852.r135138

**Published:** 2022-05-03

**Authors:** Manushak Avagyan

**Affiliations:** 1 International Agency for Research on Cancer, Lyon, France; 2 Hematology Center after Prof. Yeolyan, Yerevan, Armenia

The manuscript presents a detailed assessment of the mRNA-1273 vaccine's safety profile in 93 cancer patients and its link to their demographics.

 In view of the paucity of knowledge on data about the safety and efficacy of the vaccines among cancer patients, this is a potentially valuable contribution to knowledge. However, the manuscript needs adjustments to be ready for indexing in F1000Research, as covered in the following specific comments.

 Overall, the work might benefit from greater literature support; consider updating with the latest publications concerning vaccination safety in cancer patients.

**Title:**

- The title is lengthy yet self-explanatory.

**Abstract:**

- The abstract and overarching objectives are clear. The methodology is not thorough and the analysis is not clear from the appropriate section. The main outcomes are clear, and the conclusions are relevant to the aim.

**Keywords:**

- Please consider including a keyword that addresses adverse reactions in addition to safety.

**Introduction:**

- The broad description section at the beginning could be improved, particularly the first six sentences, which should be shortened and summarized into 2-3 sentences and present more about the characteristics of oncology patients, as well as a presentation about other vaccine-related information among cancer patients.
- The use of a hyperlink at the end of the introduction does not seem proper by highlighting one sentence.
- There is a need for references for the pre-last phrase concerning a few studies indicating vaccination safety among cancer patients.
- Consider removing the patient count from the final line of the introduction, while describing the aim of the study.

**Methods:**

- Move the ethical considerations to the end of the methods section.
- Please specify the time period under investigation. The methodology section (it's clearly specified in the abstract) does not specify which dates were verified for recruitment since there are dates in the middle of the paragraph discussing data collecting for additional variables.
- In the methods section, please define the term "heavily treated."
- If you find it feasible, an extra step for the study would be to use a logistic regression model and evaluate the model's fit to the data.

**Results:**

- The reported findings are based on the analysis mentioned in the methodology section.
- Having subheadings in the results section can improve.
- Consider mentioning the full date in the first sentence of the results section.
- Page 7:
  a. The second paragraph, which discusses the relationship between various doses and the subsequent systemic response, might be articulated clearly in the first opening phrase.
  b. The presentation of the findings in the third paragraph might be improved.
  c. In the fourth paragraph, the first two phrases need clarification.

**Discussion:**

- In the discussion section, only three new references are provided, and further comparisons and references are needed; this might be updated with new research released in 2022.

**Tables:**

- In Table 1, the word "SD" appears twice in the second row.
- Table 2 might be improved by deleting unnecessary information generated during analysis and presenting a clearer and more focused table, with the main values being OR, P, and CI, and the rest could be discarded.

Is the work clearly and accurately presented and does it cite the current literature?

Partly

If applicable, is the statistical analysis and its interpretation appropriate?

I cannot comment. A qualified statistician is required.

Are all the source data underlying the results available to ensure full reproducibility?

Yes

Is the study design appropriate and is the work technically sound?

Yes

Are the conclusions drawn adequately supported by the results?

Yes

Are sufficient details of methods and analysis provided to allow replication by others?

Yes

Reviewer Expertise:

Childhood cancer, cancer epidemiology, childhood cancer registration, cancer registries.

I confirm that I have read this submission and believe that I have an appropriate level of expertise to confirm that it is of an acceptable scientific standard, however I have significant reservations, as outlined above.

# Response

**Published:** 2022-07-20

**Authors:** Javier David Benitez Fuentes

**Affiliations:** San Carlos University Hospital, Spain

In the 2nd version, I am submitting the following has been addressed:

 Abstract:

- The abstract and overarching objectives are clear. The methodology is not thorough and the analysis is not clear from the appropriate section. The main outcomes are clear, and the conclusions are relevant to the aim.
**Response**: Methodology and results description has been improved.

Keywords:

- Please consider including a keyword that addresses adverse reactions in addition to safety.
**Response**: Done 

Introduction:

- The broad description section at the beginning could be improved, particularly the first six sentences, which should be shortened and summarized into 2-3 sentences and present more about the characteristics of oncology patients, as well as a presentation about other vaccine-related information among cancer patients.
**Response**: Done 
- The use of a hyperlink at the end of the introduction does not seem proper by highlighting one sentence.
**Response**: Editors from the journal suggested this format. 
- There is a need for references for the pre-last phrase concerning a few studies indicating vaccination safety among cancer patients.
**Response**: Done
- Consider removing the patient count from the final line of the introduction, while describing the aim of the study.
**Response**: Done 

Methods:

- Move the ethical considerations to the end of the methods section.
**Response**: Done
- Please specify the time period under investigation. The methodology section (it's clearly specified in the abstract) does not specify which dates were verified for recruitment since there are dates in the middle of the paragraph discussing data collecting for additional variables.
**Response**: Done
- In the methods section, please define the term "heavily treated."
**Response**: Done
- If you find it feasible, an extra step for the study would be to use a logistic regression model and evaluate the model's fit to the data.
**Response: **Clarified by the biostatistician 

Results:

- The reported findings are based on the analysis mentioned in the methodology section.
- Having subheadings in the results section can improve.
**Response**: Done
- Consider mentioning the full date in the first sentence of the results section.
**Response: **Done
- Page 7:
  a. The second paragraph, which discusses the relationship between various doses and the subsequent systemic response, might be articulated clearly in the first opening phrase.
**Response: **Done
  b. The presentation of the findings in the third paragraph might be improved.
**Response: **Done
  c. In the fourth paragraph, the first two phrases need clarification.
**Response: **Done

Discussion:

- In the discussion section, only three new references are provided, and further comparisons and references are needed; this might be updated with new research released in 2022.
**Response: **Done

Tables:

- In Table 1, the word "SD" appears twice in the second row.
**Response: **Erased
** **
- Table 2 might be improved by deleting unnecessary information generated during analysis and presenting a clearer and more focused table, with the main values being OR, P, and CI, and the rest could be discarded.
**Response: **Done
